# Global Review of Dairy Recommendations in Food-Based Dietary Guidelines

**DOI:** 10.3389/fnut.2021.671999

**Published:** 2021-05-25

**Authors:** Kevin B. Comerford, Gregory D. Miller, Amy C. Boileau, Stephanie N. Masiello Schuette, Janice C. Giddens, Katie A. Brown

**Affiliations:** ^1^California Dairy Research Foundation, Davis, CA, United States; ^2^National Dairy Council, Rosemont, IL, United States

**Keywords:** food-based dietary guidelines, dietary patterns, food groups, nutrition, dairy

## Abstract

At present, there are ~100 countries with national food-based dietary guidelines. While the intent of these guidelines is to inform national-level dietary recommendations, they also tie into global health and sustainable development initiatives, since diet and nutrition are linked to outcomes for all 17 United Nations Sustainable Development Goals. Therefore, key messaging in food-based dietary guidelines plays an important role in both national and global health efforts. However, this type of national-level dietary guidance is not standardized and varies considerably from country to country, and from food group to food group. The main objective of this review is to provide a novel look at dairy food group messaging within global food-based dietary guidelines, focusing specifically on nutrient-based and health-based messaging. Dairy-based messaging from 94 national food-based dietary guidelines was reviewed and grouped by region, with an emphasis on messaging regarding dairy's contribution to nutrients of public health concern for both underconsumption and overconsumption. The results showed that most nutrient-based dairy messaging relating to underconsumption was focused on calcium, followed by vitamin D, iodine, potassium, and protein; whereas messaging related to overconsumption was focused on saturated fat, added sugars, and salt. Health-based messaging specific to dairy food intake typically coalesced around three types of health outcomes: (1) bone, teeth, and muscle, (2) cardiometabolic, and (3) gut and immune. Although a fundamental concept of food-based dietary guidelines is to provide dietary guidance in a manner that is both “food-based,” and in the context of “dietary” patterns, most food-based dietary guidelines still express the health value of dairy foods (and potentially other foods groups) solely in terms of their nutrient content – and often times only in the context of a single nutrient (e.g., calcium).

## Introduction

Governments around the world have provided science-based dietary advice for more than a century, continually updating their recommendations to prevent nutrient deficiencies, reduce chronic disease risk, and improve human health (1). Despite these efforts, global trends in the triple burden of malnutrition (the simultaneous occurrence of undernutrition, micronutrient deficiencies, and overweight or obesity) and non-communicable disease (NCD) continue to rise. These largely preventable diet-related health outcomes are major factors inhibiting many countries' ability to achieve their health and sustainability targets, including the United Nations (UN) Sustainable Development Goals (SDGs) (2, 3).

Food-based dietary guidelines (FBDGs) became more prevalent following the 1992 International Conference on Nutrition (ICN), co-organized by the World Health Organization (WHO) and Food and Agriculture Organization (FAO) (4). A primary outcome from ICN was the recommendation that dietary guidelines shift from a nutrient- to a food-based approach since dietary decisions are generally based on foods rather than nutrients. In 1996, the WHO and FAO co-wrote a report on the development and implementation of national FBDGs (5). This report acknowledged that a wide range of dietary patterns can be consistent with good health, and that there is need for diversity in FBDGs since dietary patterns differ from country to country, as well as among distinct geographic or socioeconomic populations within the same country.

The major way in which key messaging on dietary diversity and healthy dietary patterns is promoted in FBDGs is through food group recommendations (6). While each food group is unique in its nutritional contributions to a dietary pattern, the underconsumption of certain food groups may be more detrimental to health than others. Among the core recommended food groups, dairy foods standout for providing an unmatched package of essential nutrients (7, 8), while also being produced year-round in nearly all regions of the world. These attributes, among others (e.g., high nutrient bioavailability, a functional food matrix, rich in shortfall nutrients), support the concept of achieving adequate consumption of dairy foods as a global public health strategy, with the potential to help reduce global disease burden.

The aim of this review is to summarize how the dairy food group is recommended within global FBDGs, focusing specifically on how its nutritional and health contributions are characterized. The FBDGs of 94 countries that had official FBDGs as of December 2020 were included in the assessment. The FAO's global FBDG repository served as the starting point for sourcing FBDGs (9). If the FBDGs posted on the FAO's site were out-of-date, then national health and nutrition agency websites were used to source up-to-date versions. Each FBDG was reviewed for dairy-based messaging. Data were then categorized into global, regional, and country-level findings. For regional-level findings. this assessment utilized the FAO's regional categorization scheme (Africa, Asia and the Pacific, Europe, Latin America and the Caribbean, the Near East, and North America) for FBDGs, using the organization's online directory (10). One exception to this was made – to classify Israel as a “Near East” country, rather than a “European” country as indicated on the FAO's website.

## Global Food-Based Dietary Guidelines

Currently, there are ~100 countries with national FBDGs (10). As more countries introduce and update FBDGs, their functions have expanded and evolved. FBDGs inform many national food and nutrition efforts, including school feeding and nutrition assistance programs, nutrition education, food labeling, and more (11). Many countries also expanded the scope of their FBDGs as well, aiming for more inclusivity of cultural relevancy, diversity of dietary preferences and needs, affordability, and environmental sustainability.

While the intent of FBDGs is to inform national dietary guidance, FBDGs also tie into global health and sustainable development initiatives, with diet and nutrition being linked to outcomes for all 17 SDGs (2). To reassert the critical role of food and nutrition in achieving the SDGs, the UN is convening its first Food Systems Summit in 2021, with the objective of galvanizing global support to transform food systems at all levels (12). Outcomes from the Food Systems Summit could have implications for national nutrition priorities, including FBDG.

## Dairy as a Foundational Food Group in Food-Based Dietary Guidelines

There is considerable diversity in FBDGs as to how food groups are categorized. Most FBDGs discuss healthy dietary patterns in terms of three to seven recommended food groups with the most common designations being fruits, vegetables, starchy staples, dairy, and protein foods (6, 13). Each food group contributes a unique spectrum of nutrients and bioactive compounds to the diet. At the same time, each group alone cannot adequately supply all of the necessary essential nutrients for optimal health. In short, food groups are complementary to each other, not equivalent.

Dairy foods are a standalone food group in many FBDGs due solely to their calcium contributions to the diet. Dairy foods are also recognized in FBDGs as a source of multiple essential vitamins and minerals and/or high-quality protein. Although mentioned less frequently in FBDGs messaging, fermented dairy products, such as yogurt and kefir, can provide health benefits beyond their essential nutrient content. They have been shown to be among the most effective dietary carriers of probiotics and have been credited with protecting various aspects of oral health, gut health, and overall immune function (14–17). Additionally, due to their high water and electrolyte content, liquid milk and dairy products can be excellent sources of hydration which can play a critical role for preventative health in countries where sanitation issues limit access to potable water. Dairy foods also provide higher levels of several emerging nutrients of interest for optimal health (e.g., choline, taurine, lactoferrin, probiotics, milk-fat globule membrane) compared to other foods groups. These nutritional, protective and preventative properties make dairy both a foundational and functional food group in many regions of the world.

## Dairy Recommendations by Region

Dairy foods are classified as a distinct food group in roughly three-quarters of FBDGs. In the countries where dairy foods are not classified as their own specific food group, they are almost always included as an integral component of the “protein” or “foods from animals” group, along with some combination of meat, poultry, eggs, fish, nuts, and/or legumes (6). In FBDGs, the dairy food group includes diverse and culturally relevant options including various forms of milks, cheeses, yogurts, kefirs, and more. While cow's milk is most commonly recommended in FBDGs worldwide, several countries recommend dairy foods from other animal sources (goats, sheep, camel, buffalo), and/or include plant-based alternatives that are a good source of protein and fortified to mimic the nutrition of cow's milk. In certain countries, dairy food guidance also consists of varying fat, sugar, protein, and sodium recommendations, as well as messaging emphasizing the intake of certain dairy products over others (e.g., fermented, unprocessed, vitamin D fortified, pasteurized, probiotic) (Table 1).

**Table 1:** Regional trends in dairy food recommendations in food-based dietary guidelines.

**Region**	**Range of recommended dairy foods**	**Range of recommended product qualities**	**Range of recommended serving frequencies**	**Range of recommended serving sizes**
Africa	Milk (from goats, camels, cows and sheep), Fermented Milk, Mursik, Amarurano, Maas, Yogurt, Cheese, Wara	Fresh, Fermented, Low-Fat, Skim, Low-Lactose, Plain or Unsweetened, Low-Salt	1–2 servings/day, 400–500 ml/day, in moderation, or daily intake	1 cup (250 ml) milk or yogurt, 28 g cheese, 30 g cottage cheese
Asia and the Pacific	Milk (from cows), Yogurt, Cheese, Curd, Calcium-Enriched Alternatives	Low-Fat, Reduced-Fat, Low-Lactose, Plain or Unsweetened, Fortified	1–4 servings/day, 300 g/day, or daily intake	1 cup (150–250 ml) milk, 100 g milk or milk product, 30 g milk powder, 100–200 g yogurt, 40 g cheese
Europe	Milk, Fermented/Sour Milk, Yogurt, Ayran, Cheese (Soft, Semi, Hard), Fresh Cheese (Quark, Fromage Frais), Cottage Cheese, Curd, Ice Cream, Vegetable Protein Drinks, Cheese Substitutes	Plain, Low-Fat, Fat-Free, Lean, Low in Saturated Fat, Low-Salt, Low-Sugar, Low-Lactose, Unsweetened, White, High in Calcium, Sour, Vitamin D Enriched, Protein-Rich, Fermented	2–4 servings/day, 250–500 ml/day, 300–450 g/day, or $\frac{1}{2}$ L daily intake	150–200 ml milk, 100–250 g yogurt, 200 g kefir or sour milk, 60–90 g cheese curd, 200 g fresh cheese, 85–200 g cottage cheese, 30–60 g ripened cheese, 2–3 cheese slices
Latin America and Caribbean	Milk (fluid, powdered; from cows, goat, buffalo), Yogurt, Cheese, Curd, Cottage Cheese, Kumis, Kefir	Low-Fat, Fat-Free, Low-Salt, Soft, White, Pasteurized, Fermented, Probiotic, Minimally Processed, Low-Sugar, Low-Lactose, Natural, Unflavored, Unsweetened	1–5 servings/day, 500 cc/day, in moderation, daily intake, or 2–3 servings/week	0.5–1 cup (200–245 g) milk, 150–245 g yogurt, 60 g cottage cheese 34 g curd, 20–30 g cheese, 2 tbsp grated cheese, 20–27 g milk powder, 1/4 cup evaporated milk,
Near East	Milk (fluid, powder), Cheese, Soy Products, Yogurt (Laban and Kushk), Yogurt Drink (Doogh), Labneh (Strained Yogurt/Soft Cheese), Cream Cheese, Ice Cream,	Low-Fat, Fat-Free, Fermented, Without Additives, White, Low-Sodium, Natural, Plain, Unflavored, Unprocessed, Vitamin D Fortified	1–4 servings/day, or daily intake	1 cup (240 ml) milk or yogurt, 30–60g cheese, 1 cup milk-based pudding, 8 tbsp labneh, 1.5 cups low-fat milk-based ice cream, 3 spoons milk powder,
North America	Milk (fluid, dry, evaporated), Yogurt, Cheese, Kefir, Frozen Yogurt, Buttermilk, Dairy Desserts, Fortified Soy Beverages and Soy Yogurt	Low-Fat, Fat-Free, Low-Lactose, Lactose-Free, Low-Sodium, Unsweetened, Fortified	2–3 servings/day	1 cup (250 ml) milk, 8 oz or 175 g yogurt, 1.5 oz or 50 g cheese

*Messaging reflects the most current FBDGs as of December 2020*.

### Africa

Seven countries (13% of countries in region) in Africa have FBDGs, with several others working toward releasing their first edition (18). With the exceptions of Namibia and Sierra Leone, dairy is its own food group in all African FBDGs. Most FBDGs recommend daily intake of low-fat dairy, though there are variations in how this amount is specified. For example, Kenya and Sierra Leone recommend “daily” intake, while Benin and South Africa provide quantitative guidance (1–2 servings per day and 400–500 ml per day, respectively). Dairy is often recognized for contributing to healthy and diverse diets. For example, both Nigeria and Sierra Leone note the importance of dietary diversity and the role that dairy foods play in supporting this.

### Asia and the Pacific

The Asia and Pacific region encompass 17 countries with FBDGs (53% of countries in region). Most FBDGs classify dairy as its own food group and have key messaging that encourage dairy food intake and/or provide specific dairy food guidance (6). The most common recommendations are for 1–2 servings/day, although Australia's and New Zealand's recommendations are higher at 2.5–4 servings per day depending on age and gender. The most common dairy messaging recommendations suggest selecting low-fat or fat-free options, consuming dairy foods for their calcium content, and for enhancing the health benefits of dietary patterns by combining dairy along with a variety of other nutrient-rich foods. Dairy foods are recommended for different reasons across the region. For example, in Bangladesh, Sri Lanka, Thailand, and Fiji, dairy is primarily recommended for bone and dental health, and in India dairy is described as “body building” and “protective.”

### Europe

Thirty-two countries (62% of countries in region) in Europe have FBDGs. Dairy foods are their own food group in the vast majority of these (6). More than half of European FBDGs recommend 2–4 servings of dairy per day, and >80% contain some form of specific dairy messaging. Messaging generally focuses on selecting low-fat and fat-free options, as well as fermented dairy foods and a variety of cheeses. Many countries emphasize dairy's calcium contributions, while only a few mention other nutrients. For example, Belgium includes messaging on achieving adequate protein, vitamin B2 and B12, while Sweden includes messaging on consuming dairy for vitamin D, Iceland for iodine, and North Macedonia for potassium.

### Latin America and Caribbean

Twenty-nine countries (88% of countries in region) in this region have FBDGs. Here, dairy foods are often categorized into a “Foods from Animals” group, rather than a dairy or protein foods grouping. Less than 40% of countries consider dairy to be its own food group, while ~30% of countries have specific messaging for dairy foods in their FBDGs (6, 13). Several countries do not specify how much dairy to consume each day. Of those that do, the recommendation is to eat dairy foods or “foods from animals” daily or weekly, but the amount varies from 1 to 5 servings. Most FBDGs recommend skim or low-fat dairy. This region emphasizes dairy and calcium intake for bone, teeth, and muscle health.

### Near East

There are currently seven FBDGs available across the Near East and all seven classify dairy as its own food group. Low-fat or fat free dairy is recommended on average between 1 and 4 times per day. However, the recommended types of products, intake amounts, frequency of intake, and acceptable alternatives vary considerably among countries (19). The most consistent dairy-related nutrition and health messaging among countries is focused on calcium and/or vitamin D intake and bone health. Several of the FBDGs from Near East countries are similar to North American dietary guidance (e.g., based on a food pyramid or food plate models), therefore several of the general dairy recommendations are similar to those found in the U.S. and Canada.

### North America

Both Canada and the U.S. have FBDGs, but only the U.S. considers dairy to be its own food group. For decades, both countries have recommended dairy foods for daily consumption (20, 21). Canada's national food guide was updated in 2019. Dairy is included in the protein food group, which is comprised of both plant and animal sources of protein-rich foods. Key dairy messages include consuming lower fat dairy products, like milk yogurt and lower sodium cheeses. Since its first publication in 1980, the U.S. Dietary Guidelines for Americans (DGA), has regarded the dairy group as one of the core elements that makes up a healthy dietary pattern for Americans (22). The 2020–2025 DGA recommends the intake of 3 servings per day as part of a 2,000-calorie “Healthy U.S.-Style Dietary Pattern,” and as part of “Healthy Vegetarian Dietary Pattern” for all populations above the age of 2 years (23). The DGA reinforces that there are several types of healthy eating patterns which are dependent on regular daily intake of dairy products – especially low-fat and fat-free dairy.

## Dairy and Nutrient-Based Messaging in Food-Based Dietary Guidelines

Although the fundamental concept of FBDGs is to provide nutrition guidance in a manner that is both “food-based,” and in the context of “dietary” patterns, many FBDGs express the health value of foods solely in terms of their nutrient content. In the case of dairy foods, dietary guidance has historically depended on its contribution to essential nutrient intake, including nutrients of concern due to underconsumption and overconsumption (Table 2).

**Table 2:** Examples of milk and dairy nutrient-based messaging in FBDGs.

**Nutrient**			**Country**	**Messaging examples**
Nutrients of concern for underconsumption	Macronutrients	Protein	Italy	^*^ “Drink a cup of milk or yogurt every day, preferably choosing the partially skimmed one, which maintains its content in calcium and proteins.”
			Panama	^*^ “[Dairy] provides vitamin B and D, and minerals like calcium and phosphorus, as well as protein, to form and maintain bones and tissue.”
			Bangladesh	“*Milk also contains protein, lactose and vitamins (especially vitamin B12) which promotes growth and proper functioning of body tissues.”*
	Vitamins	Vitamin A	Belize	“*Milk is recognized as contributing vitamins A, B and calcium to the diet.”*
			United States	“*The dairy group contributes many nutrients, including calcium, phosphorus, vitamin A, vitamin D (in products fortified with vitamin D), riboflavin, vitamin B12, protein, potassium, zinc, choline, magnesium, and selenium.”*
		Vitamin B2 (Riboflavin)	Belgium	“*If the intake is <250 ml/day, care should be taken to provide calcium, vitamin B2, vitamin B12 and protein from other foodstuffs.”*
		Vitamin B12	Bangladesh	“*Milk also contains protein, lactose and vitamins (especially B12), which promotes growth and proper functioning of body tissues.”*
		Vitamin D	Fiji	“*Milk provides key nutrients such as calcium and vitamin D.”*
			Qatar	“*Choose vitamin D fortified milk.”*
			Sweden	“*Choose low-fat, unsweetened products enriched with vitamin D.”*
	Minerals	Calcium	India	“*Milk, curds and nuts are rich sources of bio-available calcium.”*
			Italy	^*^ “The characterizing element of this group [milk and dairy foods] is the high content of calcium in an easily absorbable form and usable.”
			Oman	“*The main source of calcium in the Omani diet is milk…Diets that include milk products tend to have a higher overall nutritional quality.”*
		Iodine	Australia	“*Milk and dairy foods are an important source of iodine in Australia.”*
			Iceland	^*^ “Most of the iodine in the diet in Iceland comes from dairy products and fish.”
			Sweden	“*Choosing salt with iodine is a good idea as iodine is needed for the metabolism. But you don't need huge quantities of salt to get enough iodine, you'll find iodine in milk and seafood as well.”*
		Phosphorus	Panama	^*^ “[Dairy] provides vitamin B and D, and minerals like calcium and phosphorus, as well as protein, to form and maintain bones and tissue.”
			Thailand	“*Milk is good for everyone. It is rich in calcium and phosphorous, which are essential for building strong bones and teeth.”*
		Potassium	North Macedonia	^*^ “Sources of potassium are found in almost every group of products, especially in vegetables, fruits, and milk dairy products.”
			South Africa	“*Milk and dairy products are excellent sources of several micronutrients, as well as being relatively low in sodium and high in potassium.”*
			United States	“*The dairy group contributes many nutrients, including calcium, phosphorus, vitamin A, vitamin D (in products fortified with vitamin D), riboflavin, vitamin B12, protein, potassium, zinc, choline, magnesium, and selenium.”*
Nutrients of concern for overconsumption	Macronutrients	Fat	Grenada	“*Change from whole milk to reduced fat, from reduced fat to low fat and from low fat to fat-free or non-fat or skimmed milk.”*
			Lebanon	“*As full-fat milk and dairy products can substantially contribute to the intake of total fat and saturated fat, low-fat and fat-free versions should be selected.”*
			Netherlands	^*^ “Low-fat and semi-skimmed dairy are preferable to full-fat dairy.”
		Sugar	Chile	^*^ “To strengthen your bones, consume low-fat and low-sugar dairy three times per day.”
			Iceland	^*^ “It is recommended to choose the most low-fat, unsweetened or low-sugar dairy products without sweeteners.”
			Kenya	“*Use milk and milk products with little or no added sugar.”*
	Minerals	Sodium	Argentina	^*^ “Choose soft cheeses over hard ones and those with less fat and salt content.”
			Bulgaria	“*Prefer milk and dairy products with low fat and salt content.”*
			Lebanon	“*Consume natural cheeses more often than processed cheeses, as the latter contain higher amounts of salt.”*

* *Translated to English*.

*Messaging reflects the most current FBDGs as of December 2020*.

### Dairy's Contribution to Nutrients of Public Health Concern for Underconsumption

Each country suffers from its own unique set of nutrient imbalances and associated disease burdens; but these are not consistently reflected in FBDGs as some countries strongly emphasize the nutrient contributions from the various food groups, while others mention few to none. For example, the DGA lists more than a dozen nutrients found in dairy foods, while most other countries only emphasize a few (Table 2). Of the 12 dairy-derived nutrients (protein, vitamin A, vitamin B2 (riboflavin), vitamin B12, vitamin D (in products fortified with vitamin D), choline, calcium, iodine, phosphorus, potassium, selenium, zinc) mentioned in global FBDGs, six are recognized as some of the most consistently underconsumed nutrients worldwide. In particular, low intakes of vitamin A, iodine, and zinc are among the most common nutrient deficiencies globally, disproportionately impacting low- and-middle income regions (24), while calcium, potassium, and vitamin D are all listed as dietary components of public health concern in industrialized regions such as North America (23). Most nutrient-based dairy messaging in FBDGs currently focuses on calcium, followed by vitamin D, iodine, potassium, and protein, whereas messaging for critically underconsumed nutrients such as vitamins A and zinc is rare. This seems like a major missed opportunity for dietary guidance and public health, as a greater focus in FBDGs on foods that contain multiples of these shortfall nutrients could be a strong focus area for policies and programming aimed at reducing the incidence of undernutrition.

### Dairy's Contribution to Nutrients of Public Health Concern for Overconsumption

Given concerns with overconsumption of certain nutrients, dairy guidance is often accompanied by reminders to avoid dairy foods rich in saturated fat, added sugars, and salt. Some of this dietary guidance has been challenged and continues to require research, especially for intake of saturated fats on health, which has consistently been shown to be largely dependent on the fatty acid characteristics (i.e., carbon chain length, and number and location of unsaturated double bonds) and not just the total quantity or ratio to other calories consumed (e.g., <10% of energy). Despite the growing body of research showing that dairy fats can be neutral or beneficial for health (25–27), most FBDGs continue to consistently recommend low-fat and fat-free products for caloric reasons. The total sugar content or type of sugar (naturally occurring vs. added sugars) in dairy foods are mentioned in ~30% of FBDGs worldwide. These recommendations generally focus on nudging toward natural sugars contained in foods over added sugars like sucrose; promoting dairy products over soft drinks; and promoting the substitution of unsweetened dairy products for sweetened products. The overall objectives of these messages vary by region, but generally have to do with improving oral health and energy balance. Approximately 20% of FBDGs also mention salt or sodium intake in their dairy messaging. These messages are generally in the context of reducing intake of high-sodium cheeses, and occur primarily in FBDGs from Europe, North America, and Latin America and the Caribbean.

## Dairy and Health-Based Messaging in Food-Based Dietary Guidelines

Health-based messaging specific to dairy food intake typically coalesces around three health outcomes: (1) bone, teeth, and muscle, (2) cardiometabolic, and (3) gut and immune (Table 3). Fewer than one-third of FBDGs contain messaging promoting dairy intake in the context of specific health outcomes. The vast majority of these focus on bone and teeth health, while a much smaller percentage address other health outcomes. This presents a major opportunity for improving global health as higher intake of dairy foods have consistently been shown to improve health by reducing the incidence of numerous nutrient deficiencies, as well as the ability to mitigate the risk for some of the most prolific and debilitating chronic diseases, including cardiovascular disease and type 2 diabetes (28–31). Notably, two recent systematic reviews focused on inflammation (which has been hypothesized to be the underlying cause of several chronic disease states), found neutral to beneficial effects of higher total dairy intake on inflammatory markers (32, 33). Similarly, a growing number of systematic reviews have also consistently found neutral to beneficial effects of dairy intake on body composition and overall cardiometabolic health – with the most beneficial effects on biomarkers such as adiposity, serum lipids, blood pressure, and insulin resistance, being attributed to fermented, fortified, and/or low-fat dairy foods (34–39).

**Table 3:** Examples of dairy and health-based messaging in FBDGs.

**Health outcome**	**Country**	**Messaging examples**
Bone, teeth, and/or muscle health	Bangladesh	“*Consumption of milk is essential for young children and adolescents for building maximum peak bone mass. Milk consumption also helps to prevent osteoporosis in later life.”*
	Sierra Leone	“*Choose beverages, e.g., dairy products such as milk, yogurt, cheese, which strengthen teeth.”*
	United Kingdom	“*Try to have some milk and dairy food (or dairy alternatives) – such as cheese, yogurt and fromage frais. These are good sources of protein and vitamins, and they're also an important source of calcium, which helps to keep our bones strong.”*
	Saudi Arabia	“*The committee decided to have the number of milk and milk products servings to be between 2 and 4 servings, to have normal growth, to reduce prevalence of rickets and osteomalacia among Saudi society.”*
	United States	“*The nutrient composition of dairy foods highlights the importance of adequate consumption. This is especially relevant for calcium and vitamin D, given that adolescents have an increased need for consumption to support the accrual of bone mass.”*
	Uruguay	^*^“Without dairy for breakfast, it's difficult to reach the three servings we need per day to maintain bone health.”
Cardiometabolic health	Finland	^*^“Abundant use of skimmed and low - fat dairy products is connected to lower hypertension, stroke and risk of type 2 diabetes.”
	North Macedonia	^*^“Consumption of milk and dairy products is also associated with a reduced risk of cardiovascular disease, type 2 diabetes, and lower blood pressure in adults.”
	Oman	“*Many cheeses, whole milk, and products made from them are high in saturated fat. To help keep blood cholesterol levels healthy, limit the amount of these foods you eat.”*
Gut and immune health	Austria	^*^“Milk does not increase the risk of asthma.”
	Costa Rica	^*^“Prefer yogurt with probiotics because it improves the bacterial flora.”
	El Salvador	^*^“When there is an intolerance to the sugar of milk (lactose) can be consumed lactose-free.”
	Romania	“*If you do not consume milk or milk products due to lactose intolerance the most useful method of benefiting from the benefits of this food is the consumption of delactose preparations.”*

* *Translated to English*.

*Messaging reflects the most current FBDGs as of December 2020*.

## Discussion

FBDGs are continually updated in response to an everchanging food-diet-nutrition-health landscape. This review provides a snapshot as of the year 2020, as to how dairy food group messaging is used in global FBDGs. This novel approach to assessing food group messaging in national, regional, and global contexts could prove useful for enabling a better understanding how factors such as culture, economics, and geography influence FBDGs.

This analysis revealed that more than 70% of FBDGs contain messaging regarding the consumption of dairy foods (6). This messaging generally focuses on basic information such as recommended options (e.g., milk, yogurt, cheese), serving sizes (e.g., 200 ml milk, 1 cup yogurt, 30 g cheese), consumption frequency (e.g., daily intake, 2–3 servings/day) (Table 1). Dairy-based messaging is more common in FBDGs from Europe, the Near East, and North America, when compared to Africa, Asia and the Pacific, and Latin America and the Caribbean. However, since each region contains a diverse set of FBDGs encompassing many unique country-level relationships with dairy foods, it is difficult to succinctly compare FBDG dairy messaging at the regional level. Some of the more interesting inter-region statistics regarding dairy foods and FBDG messaging are also difficult to decipher as roughly 30% of countries, especially those from Latin America and the Caribbean, either do not mention dairy in their FBDGs or end up including dairy in their “protein foods” or “foods from animals” groups. In many of these cases, dairy foods are not specifically referenced, making it unclear as to how dairy food intake is viewed in these countries (6).

In terms of nutrient and health messaging, the majority of FBDGs focus on dairy's contributions to adequate calcium intake and its benefits for bone and teeth health. Other common messages simply focus on dairy's contributions of one or more nutrients (e.g., protein, calcium, iodine, and vitamin D in fortified products), to a healthy dietary pattern without providing any specific health associations. Therefore, only a few FBDGs include language describing the ability of adequate dairy intake to reduce the risk for various forms of malnutrition and chronic disease (Table 3). This is a major missed opportunity for improving public health, and hopefully one that can be remedied in future national and international initiatives and action plans. Unfortunately, taking advantage of this opportunity will be challenging as there does not appear to be a linear correlation between dairy messaging in FBDGs and population-level dairy intake. A prime example of this issue is exhibited in the U.S., which produces some of the most comprehensive dairy messaging of any national FBDGs, but at the same time, has over 90% of its population failing to meet the recommended levels for dairy food intake (23).

While there is a growing body of evidence showing that increasing dairy food intake could be part of the solution to help reduce the global disease burden (37, 40), there is also a growing body of evidence investigating the role of dairy foods as as sustainable source of nutrition (41–46). These investigations have so far returned conflicting results, which largely depend on the specific foods or food groups being investigated (e.g., dairy, beef, or all animal-sourced foods), the type of modeling used (e.g., life cycle assessment, enviromental impact analysis, global foodsystem modeling), the breadth of analysis performed (e.g., a single indicator vs. multiple indicators), the specific outcomes being measured (e.g., carbon footprint per kg of food vs. per 100 kcal of food vs. per g of nutrient in a food) and where the food is produced (e.g., high-income vs. low-and-middle income countries). When compared to research on healthy diets alone, research on healthy and sustainable diets is much more complex, encompassing four interconnected sustainability domains (health, economics, society, and the environment) (47), as well as several cross-cutting factors such as innovation, food safety, power, equity, ethics, and the passage of time (48). Many of these sustainability factors are both difficult to tease apart and to measure. However, when international science-based groups like the FAO and Global Alliance for Improved Nutrition (GAIN) attempt to take all of these factors into consideration, they conclude that animal-sourced foods, such as dairy foods, should be included in healthy and sustainable diets as they play crucial roles in the nutritional, social, and economic stability of individual countries, epecially in low- and middle-income countries, as well as playing important roles in global food security and poverty reduction (49–52). In contrast to these recommendations, there are several international expert-led publications and intiatives which come to very different conclusions and aim to influence FBDGs for the purpose of reducing the consumption of animal-source foods primarily for environmental reasons (53, 54). While these environmental concerns are both legitimate and require urgent attention, their over-attribution to animal agriculture, and in particular to dairy cows, is often misguided and deserving of much more nuance than they are usually given in discussions of sustainable diets (55, 56). For one, while agriculture (including both plant and animal agriculture, as well as forestry and other related land uses) contributes to about one-quarter of global greenhouse gas (GHG) emissions, only around half of this value is attributed to animal agriculture (57, 58). Furthermore, less than two-thirds of the GHGs from animal agriculture can be attributed to cattle, and less than half of that amount is attributed to dairy cows – resulting in dairy cows contributing to between ~2.2–2.8% of global GHG emissions (58, 59). Secondly, about half of these agriculutural GHG emissions come from methane, a short-lived GHG which when coming from plant or animal sources is considered as part of a biogenic carbon cycle that pulls carbon from the air (i.e., uses recycled carbon that is already in atmospheric circulation). This cycle, and its environmental effects, differ considerably compared to the methane released from fossil fuels, which release carbon into atmospheric circulation that has been sequestered deep underground for millenia (60). These two different methane sources should not be considered equivalent in discussions of their warming potential, since agricultural GHGs are essentially coming from a constantly recycled source of carbon, whereas the GHGs from transportation, manufacturing, electricity and heating sectors are dependent on processes that are constantly releasing “new” carbon into the atmosphere. Third, recommendations for reducing animal-sourced food messaging in FBDGs for environmental reasons tend to overlook the fact that some of the most nutrieconomic food options (e.g., dairy foods such as milk and yogurt) (7, 41) are already being massively underconsumed around the world, resulting in global deficiencies in some of the exact nutrients [e.g., calcium, iodine, potassium, protein, and vitamin D (in products fortified with vitamin D)] that these foods provide (23, 24, 56, 61). Therefore, further reductions in the availability of these shortfall nutrient-rich foods would likely lead to significant increases in unecessary suffering and healthcare costs from malnutrition and chronic diseases (62, 63). Fourth, many of the environmental sustainabilty arguments that call for reducing animal-source foods in global diets often omit the trade-offs and unintended consequences of significantly reducing animal agriculture from food systems, which would negatively impact the health, livelihoods, social structures, and traditions of billions of people worldwide who depend on animal agriculture for income, assets, land management, and sustenance (56, 64–67). In the end, the conversations regarding animal-sourced foods, such as dairy foods, in FBDGs are multifaceted and constantly changing, and should not be distilled down to a single factor such as GHGs, nutrients, money, or traditions. All of these multidimensional issues, and more will need to be considered in future FBDGs if they are to represent both healthy and sustainable diets and align with the SDGs.

What is often lost in translation when moving from the research literature to FBDGs, is that the dairy foods offer an unmatched package of essential and bioavailable nutrients, numerous functional properties (e.g., probiotics, immunoglobulins, bioactive peptides), and the ability to reduce key risk factors for some of the world's most common and deadly diseases (e.g., micronutrient deficiencies and cardiometabolic diseases). Dairy foods have also been recognized globally for their accessibility, affordability, and acceptability, and while these considerations consistently make it into publications regarding their importance to the SDGs (68–70), they rarely make it into FBDGs. Although most of dairy's contributions to healthy and sustainable dietary patterns are not captured in current FBDGs, they have tremendous potential for improving public health. As FBDGs evolve to better align with the SDGs' nutrition and health goals, it will be imperative that they continue to shift their dairy recommendations beyond a focus on individual nutrients toward a more holistic focus on the beneficial roles that these foods (especially fermented, fortified, and low-fat options) can play in reducing multiple forms of malnutrition and chronic disease.

## Author Contributions

KB and JG: conceptualization. KC: original draft preparation. All authors contributed to review, editing, and subsequent draft preparation. All authors have read and agreed to the published version of the manuscript.

## Conflict of Interest

GM, KB, SS, JG, and AB work for National Dairy Council. KC collected consulting fees for developing this manuscript.
